# A High Throughput and Unbiased Machine Learning Approach for Classification of Graphene Dispersions

**DOI:** 10.1002/advs.202001600

**Published:** 2020-08-25

**Authors:** Md. Joynul Abedin, Titon Barua, Mahdokht Shaibani, Mainak Majumder

**Affiliations:** ^1^ Nanoscale Science and Engineering Laboratory (NSEL) Department of Mechanical and Aerospace Engineering Monash University Clayton VIC 3800 Australia; ^2^ ARC Research Hub on Graphene Enabled Industry Transformation Monash University Clayton VIC 3800 Australia; ^3^ Vimmaniac Ltd. 83/A, Sugandha, Panchlaish Chittagong 4203 Bangladesh

**Keywords:** graphene oxide quality control, fake graphene detection, graphene quantification, machine learning, quantitative polarized light microscopy

## Abstract

Significant research to define and standardize terminologies for describing stacks of atomic layers in bulk graphene materials has been undertaken. Most methods to measure the stacking characteristics are time consuming and are not suited for obtaining information by directly imaging dispersions. Conventional optical microscopy has difficulty in identifying the size and thickness of a few layers of graphene stacks due to their low photon absorption capacity. Utilizing a contrast based on anisotropic refractive index in 2D materials, it is shown that localized thickness‐specific information can be captured in birefringence images of graphene dispersions. Coupling pixel‐by‐pixel information from brightfield and birefringence images and using unsupervised statistical learning algorithms, three unique data clusters representing flakes (unexfoliated), nanoplatelets (partially exfoliated), and 2D sheets (well‐exfoliated) species in various laboratory‐based and commercial dispersions of graphene and graphene oxide are identified. The high‐throughput, multitasking capability of the approach to classify stacking at sub‐nanometer to micrometer scale and measure the size, thickness, and concentration of exfoliated‐species in generic dispersions of graphene/graphene oxide are demonstrated. The method, at its current stage, requires less than half an hour to quantitatively assess one sample of graphene/graphene oxide dispersion.

## Graphical abstract

Conventional microscopy has difficulty identifying the size and thickness of graphene and graphene oxide stacks due to poor contrast. Here, using a contrast mechanism rooted on the optical anisotropy of these materials, thickness‐specific rich information is obtained, and further coupling the pixel‐by‐pixel information with an unbiased ML algorithm, these materials are classified and quantified in liquids.

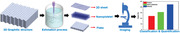

## 1 Introduction

Since the discovery of graphene in 2004,^[^ ^1^ ^]^ 2D atomic layers of graphene/graphene oxide (G/GO) have shown immense potential in various fields because of their exceptional physical and chemical properties. However, the translation of this potential to real‐life, and usable products have been slow. One of the reasons behind this has been the lack of reliability, and consistency of what is commercially often available as “graphene.” A recent study^[^ ^2^ ^]^ on commercially available graphene obtained from 60 different companies revealed that <10% of the flakes are actually single to few layer graphene sheets, and rest are flakes or graphitic structures. Currently, the most widely used method of producing G/GO sheets is the liquid phase exfoliation (LPE) process due to simplicity of the synthesis process and production at scales.^[^ ^3^, ^4^ ^]^ In this process, the single layer sheets are stripped from its 3D counterpart such as graphite (Gt), graphite oxide (GtO) film or expanded graphite by shear‐forces. The exfoliation process is highly statistical in nature and results in the formation of stacks of varying numbers of 2D atomic layers^[^ ^2^ ^]^ which can practically range from one‐two layers to larger than hundred atomic layers depending on processing conditions and solvation characteristics of the dispersing medium.^[^ ^3^ ^]^ Although there has been a strong emphasis on standardization guidelines of graphene materials,^[^ ^5^ ^]^ there is virtually no way to monitor the fundamental unit process of exfoliation, product quality varies from laboratory to laboratory and from one manufacturer to other. As a result, discrepancies are often observed in the reported property‐performance characteristics, even though the material is claimed to be G/GO.^[^ ^5^ ^]^ There is an urgent need to overcome this issue to ensure global reproducibility of the performance and accelerate the current slow‐growth of G/GO translation as mentioned by Kauling et al.^[^ ^2^ ^]^

The existing imaging tools for visualization of the exfoliated 2D sheets such as atomic force microscopy (AFM), scanning electron microscopy (SEM), and transmission electron microscopy (TEM) require extensive pre‐processing such as by repeated centrifugation, and membrane dialysis. Subsequently, the samples are flat dried on the substrate for imaging,^[^ ^6^ ^]^ discarding the thicker materials and excluding any possible 3D structures such as face‐to‐face stacking, wrinkling, rippling, and tortuosity in the solvents. These methods are no doubt useful for the visualization of the sheets, but are time consuming, and are not suitable for visualization of these materials when dispersed in a liquid, and as emphasized by Bøggild^[^ ^5^ ^]^—this remains a challenge in the field. The photon absorption capacity of a single graphene sheet is ≈2.3% over the visible spectrum, making it impossible to discern single to few‐layer G/GO sheets in a typical brightfield microscopy,^[^ ^7^ ^]^ although micron‐level thick graphite or graphite‐oxide flakes can be distinguished.

Most 2D materials exhibit anisotropy of the index of refraction—the large in‐plane refractive index results in a slow‐axis and the small out‐of‐plane refractive index gives rise to a fast‐axis of light propagation within the same material. When polarized light travels through these materials, it splits into two orthogonal waves that propagate with different velocities giving rise to contrast which is quantifiable in terms of optical retardance (*R*)^[^ ^8^ ^]^

(1) $$\begin{array}{c} R=\Delta n\times t \end{array}$$

The differential refractive index or birefringence (∆*n*) is an intrinsic optical property of the material. The retardance is amplified due to the increase in thickness (*t*). Since the solvents used in the exfoliation process are not optically birefringent, imaging methods such as polarized optical microscopy (POM) enhances the thickness‐dependent contrast of these materials. Because the birefringent species has orientation dependent optical retardance, quantitative imaging of these materials is not possible in a conventional POM.^[^ ^9^ ^]^ To overcome this issue, we have used a quantitative polarized optical microscope (qPOM) known as “LC‐PolScope” which employs both the circularly‐ and elliptically polarized lights with the help of variable retarders controlled by electro‐optical devices.^[^ ^10^ ^]^ This enabled us to image the samples regardless of their orientation, thus facilitating quantification of their retardance and slow‐axis orientation at every pixel of the image simultaneously without mechanical rotation of the specimen. This technique generates good contrast for the visualization and quantification of sub‐nanometer level birefringent species such as cell spindles,^[^ ^9^ ^]^ chromonic crystals,^[^ ^11^ ^]^ nanoparticles,^[^ ^12^ ^]^ and colloids,^[^ ^13^ ^]^ and has tremendous potential in characterizing 2D materials. Utilizing contrasts in their brightfield and cross‐polarized optical features and processing them through an unsupervised machine learning algorithm,^[^ ^14^ ^]^ we report a technique for detecting, classifying, and quantifying the exfoliated‐layered‐materials directly without any additional marker and as material dispersed in a solvent. We note that machine learning tools have been used to analyze the features of human cells,^[^ ^15^ ^]^ and brain^[^ ^16^ ^]^ which led to the discovery of rare cell types, detection of cancerous cell, and establish cause–effect relationships in psychotic disorders, but has never been used in addressing the problem of identifying exfoliated‐species. We further demonstrate that undertaking a pixel‐by‐pixel analysis we can further measure the size, thickness, and concentration of these exfoliated‐species.

## 2 Results

### 2.1 The LPE Process and Difficulty of Quantification with Existing Imaging Techniques

To bring the limitations of conventional microscopy into context, dried GtO films (Figure S1, Supporting Information) were exfoliated in water at three different shearing speeds for a period of ≈30 min—a process which is typical to LPE. The samples were imaged in elliptically polarized light field which shows the expected variation of degree of exfoliation (Figure S2, Supporting Information) indicated by an enhancement of brightness in the samples produced with increased shearing speed. AFM on these samples using typical sample preparation methods clearly demonstrates the existence of single layer 2D sheets; however, the sample preparation needs are extensive, measurement time is lengthy with possibility of human‐bias, and reveals very little about the true, statistical variation of various particle‐types produced by LPE.

### 2.2 Combined Quantitative Polarized Light Microscopy (qPOM) and Unsupervised Machine Learning Method: A Unique Analytical Platform

**Figure 1:**
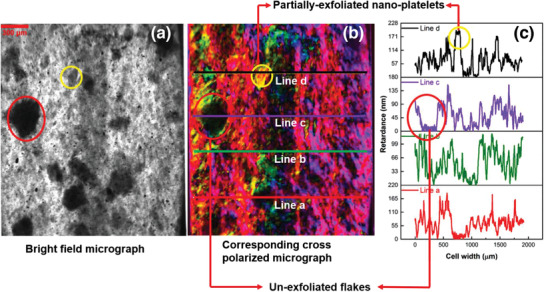
Representative optical images of LPE GO reveals distinguishable optical features. a) The transmitted intensity of the LPE‐processed materials under brightfield microscopy shows dark nontransmitting regions indicative of unexfoliated particles and medium transmitting regions indicative of partially exfoliated particles. b) Corresponding cross‐polarized micrograph imaged by the qPOM reveals the variation of optical phase retardation and slow‐axis orientation of these particles. c) Line profiles of retardances across features of interest.

**Figure 2:**
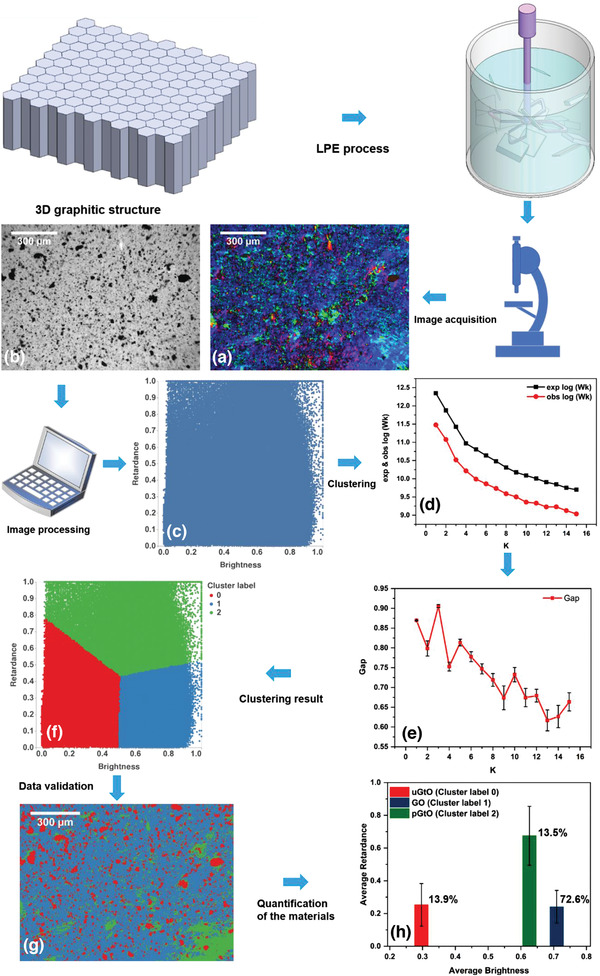
Framework for quantitative analysis. The input consists of: a) brightfield and b) cross‐polar micrographs. c) Pixel‐by‐pixel distribution of two principal components brightness and retardance for the measured datasets. d) The expected and observed sum‐of‐squared error functions dropped monotonically with the number of clusters. e) The Gap‐statistics method reveals the optimal number of clusters. The error bar represents standard deviation (SD). f) The features are clustered into three different subgroups labeled by 0, 1, and 2. g) The clustered features were labeled into the input images, which classified different species and validated the clustering process. h) The algorithm generated detailed statistics (mean ± SD, sample size) of each cluster to quantify the proportion of uGtO flakes, pGtO nanoplatelets and 2D GO sheets in the sample.

Using optical features from brightfield and polarized light microscopy, and then finding clusters and patterns in the datasets of brightness and corresponding retardance, we offer a remarkably automated, unbiased, and rapid method to overcome this critical challenge (**Figure** **1**). Central to the method, is our use of qPOM—a microscopy technique enabling the visualization and pixel‐by‐pixel quantification of even sub‐nanometer level retardance which is not possible with conventional POM techniques. These features are shown in Figure 1. The low‐transmitted intensity regions of the brightfield image features unexfoliated graphite oxide (uGtO) flakes (circled in red). The uGtO flakes are the thickest particles in the dispersion and are opaque in brightfield and isotropic in cross‐polar field. These flakes settle flat on the *x*–*y* plane, which results in perpendicular orientation of the slow‐axis with respect to the optic axis, with virtually no measurable retardance in the cross‐polar images. Compared to uGtO, the partially exfoliated graphite oxide (pGtO) nanoplatelets are approximately tens of nm thick and show higher transmittance. It can be observed that the pGtO nanoplatelets feature very high optical retardances, arguably amplified by their thicknesses (encircled in yellow). This is consistent with the observation of liquid crystalline properties of layered 2D materials in water^[^ ^17^ ^]^ and other polar solvents.^[^ ^18^ ^]^ The remaining regions are characterized by the high transmittance in the brightfield micrograph with relatively uniform and small retardance arising from the self‐aligning nature of the 2D sheets. The self‐aligning behavior of 2D sheets in water and other polar solvents have been reported by us^[^ ^10^, ^12^ ^]^ and others.^[^ ^19^ ^]^ The absence of anisotropic materials would demonstrate a featureless or isotropic area in the cross‐polarized micrograph. However, it is noticeable that local sub‐nanometer retardances are also measurable from what may appear as isotropic (dark) in the cross‐polarized micrograph. The measurement of retardance in isotropic dispersions was demonstrated in our recent publication^[^ ^10^ ^]^ and is an essential element in our ability to classify and quantify exfoliated species of their sizes, shapes, and thicknesses. To maximize the information one can generate from hundreds of images and large numbers of samples in a fast, and efficient manner we developed an unsupervised machine learning algorithm to identify data clusters of similar nature, and then use image analysis to quantify the proportions of each cluster. The analytical flow‐scheme is shown in **Figure** **2**. The algorithm is fast, computationally less intensive and can give a final decision within 14 mins for a single dataset of 1936 × 1216 resolution.

**Figure 3:**
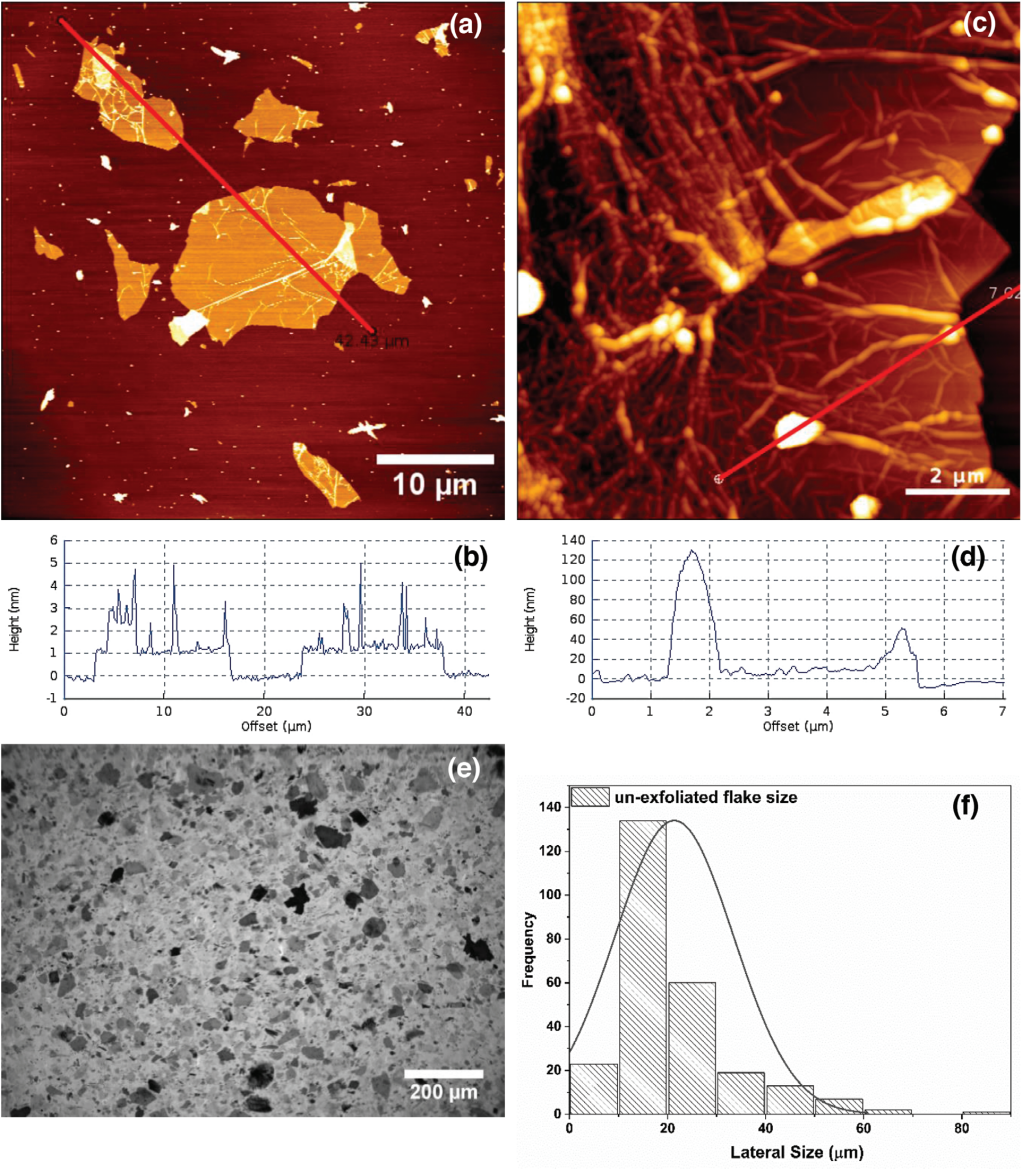
Verification by established method. Materials from three different types of clusters are imaged by AFM and polarized light microscopy. AFM imaging revealed the size and thickness of the a,b) well‐exfoliated sheets and c,d) partially exfoliated nanoplatelets. e,f) The sizes of the unexfoliated flakes were determined from the elliptically polarized light image as AFM had limitations toward measurement of very thick samples. The solid line is a normal fit of the data distribution.

We used the single‐channel 2D feature images of the principal components (brightness and retardance) with information encoded in 64‐bit floating points, and then normalized the data such that all values lie between 0 and 1 (Figure 2c). The extracted optical features are then assembled into *n* × *d* matrix, where *n* is the number of pixels, and *d* (=2) is the number of principal components. Thereafter, we employed an unsupervised statistical learning method^[^ ^20^ ^]^ widely known as *K*‐means to find similar features among the data sets in an iterative method. *K*‐means is part of the broad spectrum of “Unsupervised Machine Learning Algorithms.”^[^ ^21^ ^]^ Unsupervised statistical learning is often used to find patterns or clusters in large datasets, where predictive learning methods would be presumptuous and the answer is unknown, particularly their usefulness is noted in biological sciences to determine patterns in big‐data sets and establish cause–effect relationships.^[^ ^21^ ^]^ However, the estimation of an optimal number of clusters in the data set is a challenging task. To address this, we applied the Gap‐statistics method,^[^ ^22^ ^]^ where an error function of the pixel data is generated and concomitantly compared with an appropriate null reference distribution of the original pixel data. The error function is the sum of squared Euclidean distances around the random cluster‐means. The null reference distribution is statistically designed based on the hypothesis that if there are no subgroups present in the pixel data set, the observed or experimental error function would follow the null or expected function.^[^ ^22^ ^]^ The error function decreased monotonically with the increase of *k* but flattened noticeably from a certain *k* onward (Figure 2d). The location of such an elbow is identified as the maximum Gap between the two functions indicating the optimal number of clusters in a data set (Figure 2e).^[^ ^23^ ^]^ It can be observed that the Gap is maximum at *k* = 3 for both the local and global error measures, which signifies that the clusters are well‐separated from each other.^[^ ^21^ ^]^ It is also worth noting that the initial number of assumed cluster‐means did not influence the optimal number of clusters in the data set (Figure S3, Supporting Information). Thus, the Gap‐statistics revealed the existence of three highly distinguishable species of layered‐materials in the LPE‐processed fluid. The clustered pixel data (from Figure 2f) are assigned to the input micrograph (Figure 2g). We then analyzed each cluster through visual inspection of the segmented areas (Figure 2g) and in terms of their mean optical features (Figure 2h). We assign the highly absorbing regions containing the flakes as cluster‐0, high retardance regions containing the nanoplatelets as cluster‐2 and highly transmitting regions as cluster‐1. Each cluster is illustrated in the input binary base and cross‐polarized micrographs (Figure S4, Supporting Information). It is worth noting from cluster‐0 that the highly absorbing flakes are grouped in this cluster. The relative pixel counts in each cluster compared with the total pixels of the image allows us to estimate the relative proportions, in this particular case, 13.9% unexfoliated flakes, 13.5% partially exfoliated nanoplatelets, and 72.6% well‐exfoliated sheets. To further validate the method, we undertook an experiment in a sample in which these three clusters were present. Shear‐exfoliated GO (sample 1) was exposed to centrifugation for 10 min at 2000 rpm in order to segregate various species (unexfoliated flakes, partially exfoliated nanoplatelets, and well‐exfoliated sheets). Three different samples were collected by pipetting out from the top, middle, and bottom regions of the centrifuged sample and analyzed by AFM and elliptical polarization, which confirmed that our classification of particle types from machine learning approaches is indeed reasonable (**Figure** **3**). We do however note that the particles in cluster‐1 (well‐exfoliated) do not necessarily mean that they are all single‐layer, but inarguably the proportions of few‐layered G/GO (Figure 3c) is rather large.

### 2.3 Application of our Method to Various Laboratory‐Based and Commercial Samples

We applied our platform to an assortment of eighteen G/GO samples eight of which were acquired from commercial sources and the rest produced in the laboratory under known and controlled processing conditions (**Table** **1**).

**Table 1:** Summary of the analysis of various G/GO samples (concentrations: samples 1–10, ≈0.2 wt%; samples 11–13, ≈0.5 wt%; samples 14–17, ≈2 wt%; sample 18, ≈0.1 wt%)

Sample	Flake [%]	Nanoplatelet [%]	2D sheet [%]
1) Shear‐exfoliated at 1000 rpm for 15 min (1k15)	24.2	31.0	44.8
2) Shear‐exfoliated at 2000 rpm for 5 min (2k5)	18.4	30.6	51.0
3) Shear‐exfoliated at 2000 rpm for 30 min (2k30)	13.9	13.5	72.6
4) Shear‐exfoliated at 5000 rpm for 5 min (5k5)	9.0	31.6	59.4
5) Shear‐exfoliated at 5000 rpm for 80 min (5k80)	0	17.3	82.7
6) Shear‐exfoliated at 1000 rpm for 15 min followed by ultrasonication for 5 mins (1k15‐S5)	0	30.2	69.8
7) Shear‐exfoliated at 1000 rpm for 15 min followed by ultrasonication for 20 min (1k15‐S20)	0	25.4	74.6
8) Shear‐exfoliated at 1000 rpm for 15 min followed by ultrasonication for 80 min (1k15‐S80)	0	15.5	84.5
9) Shear‐exfoliated at 1000 rpm for 15 min followed by ultrasonication for 120 min (1k15‐S120)	0	0	100
10) Commercial graphene oxide Sigma, product 795534	0	0	100
11) Commercial graphene	51.9	10.5	37.6
12) Commercial graphene 11 shear‐exfoliated at 5000 rpm for 30 min	19.0	2.0	79.0
13) Commercial graphene	47.9	16.4	35.7
14) Commercial graphene	68.0	0	32.0
15) Commercial graphene	79.0	0	21.0
16) Commercial graphene	45.0	0	55.0
17) Commercial graphene	31.2	0	68.8
18) Commercial graphene Sigma, product 799092	24.0	0	76.0

**Figure 4:**
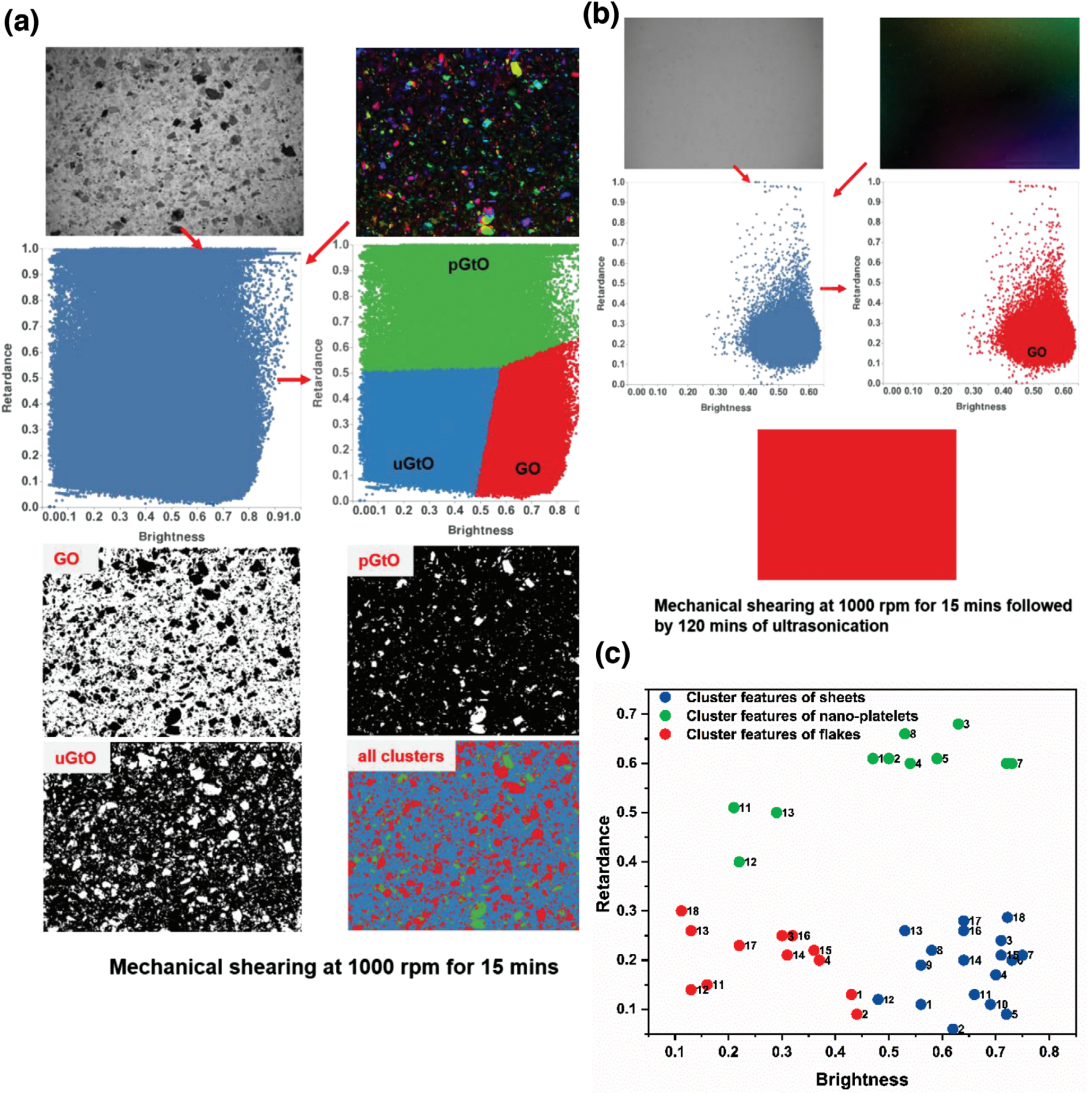
Application of our method to various laboratory‐based and commercial G/GO samples. a) Low‐speed shearing produced materials with a broad distribution of brightness and retardance indicating all sorts of materials (flakes, nanoplatelets, and sheets) are present in the sample which is revealed via a clustering process. b) Low‐speed shearing followed by a long period of ultrasonication removed features with low brightness and high retardance resulting in a single optimal cluster of exfoliated‐species. c) Well‐separated clusters exist in all samples irrespective of the source or method of exfoliation.

Selected examples from the (sample 1 and sample 9) data‐processing steps are shown in **Figure** **4a,b**. It is worth noting that in all of these samples, regardless of the source and method of exfoliation, the statistically determined clusters can be distinguishably classified in the 2D feature plots (Figure 4c), suggesting that the data could be used as training sets to classify exfoliated‐features in supervised algorithms. This method also shows the yields of exfoliation (Figure S5, Supporting Information) among various samples. Being able to quantify some commonly known and practiced methodologies in graphene production such as the increase in the yield of 2D sheets with shearing time (sample 2 vs sample 3), and shearing speed (sample 2 vs sample 4), are crucial indicators of the usefulness of our method for in situ process monitoring approaches.

A more remarkable example is shown in Figure 4b—a sample produced by low speed shearing transforms to a single cluster of exfoliated‐species identified to be 2D sheets only after long periods of ultrasonication (sample 9) and has features similar to commercial GO (sample 10). GO sheets produced from GtO flakes by similar LPE methods reported 50–80% yield,^[^ ^3^, ^24^ ^]^ which is in good agreement to the yield determined by this method. It is also noticeable that none of the commercial graphene samples show a single cluster of 2D sheets but large quantities of flakes which is consistent with recent reports.^[^ ^2^ ^]^ The results of selected commercial graphene samples are provided in (Figures S6–S8, Supporting Information).

### 2.4 Intracluster Data Analysis

**Figure 5:**
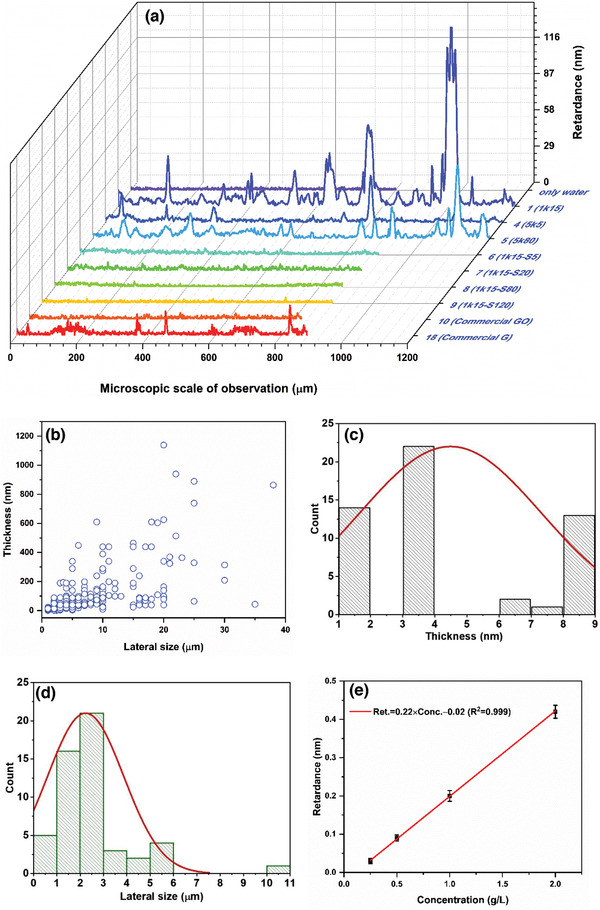
Characteristics of clusters. a) Retardance distribution analysis can provide thickness and size estimation of nanoplatelets and 2D sheets. b) Lateral size and thickness distribution of nanoplatelets and 2D sheets in sample 1. c) Thickness and d) lateral size distribution of the nanosheets containing less than ten layers are plotted to demonstrate the measurement capacity of this technique. The solid lines are the normal fits of the data. e) Correlation between concentration and mean retardance obtained from the cluster of sheets. The error bar represents SD, which is calculated from five sets of clustering data for each concentration.

The universal compensator used in this imaging method can measure the optical retardance of the birefringent specimen regardless of its orientation (Figure S9, Supporting Information) and in a further processing, by combining the circular and elliptical polarizations of that specimen algorithmically, it estimates an orientation‐resolved global retardance that varies linearly with the specimen thickness. The retardance profiles of various samples containing nanoplatelet and 2D sheet are illustrated in **Figure** **5**a. Using the average birefringence (∆*n*) of these materials which is estimated to be – 0.2 at *λ* = 546 nm, ^[^ ^10^, ^25^ ^]^ we have calculated the thicknesses of the nanoplatelet and 2D sheet from these retardance data using equation (*R* = Δ*n* × *t*) as shown in Figure 5b,c. In particular, Figure 5c shows the thickness distribution of few‐layered species (less than 10 layers) in the sample. By conducting local analyses of the spatial variation of retardance (*X*‐axis of Figure 5a), we have also estimated their corresponding lateral sizes of the 2D sheets as shown in Figure 5d. The distribution of lateral size and thickness of all the nanoplatelets and 2D sheets obtained from this sample are provided in histograms (Figure S10, Supporting Information). The average lateral size and thickness of the nanoplatelets in sample 1 are ≈18.5 ± 1.5 µm and ≈126.3 ± 37 nm, respectively. The upper limit of the retardance of this method is 273 nm, estimating a lower bound of the flake thickness ≈1.3 µm, whereas the upper bound is the initial thickness of the film ≈1 mm. The relative absorbance of this cluster in the brightfield image indicates the relative thicknesses of the flake. A much sought‐after information is the concentration of the 2D sheets in the solution phase. Based on the mean value of the retardance in the 2D sheet‐cluster (cluster‐1), we have established an experimental correlation (*R* = 0.22*C* − 0.23) between the retardance (*R*) and the concentration (*C*) for well‐exfoliated GO (Figure 5e).

## 3 Discussion

The technique is most suitable for samples containing nanoplatelets and flakes with a total concentration varying between 0.02 and 2 wt% solids. There is no significant limitation on higher concentrations, as they can be imaged in diluted form (e.g., sample 13 was diluted from 2 to 0.5 wt%). GO (sample 5) was imaged at three different concentrations (2, 1, and 0.5 g L^−1^) and then processed for quantification by this method. The average proportion of the sheet was determined to be ≈77.80% with relatively small standard deviation of ± 3.7%. Similarly, commercial graphene (sample 18) was imaged at three different concentrations (1, 0.5, and 0.25 g L^−1^) and then processed by this method estimated ≈66.67 ± 7% sheets in the sample (Figure S11, Supporting Information). We had some practical imaging difficulties in low concentrations (<0.1 wt%) because the species in the liquid flow randomly during the process of acquiring the brightfield and retardance images. Usually there is a small interval of ≈30 s in acquiring these two images simply because the polarizers have to be detached. A possible solution to this is to use elliptically polarized light image instead of brightfield image (elliptically polarized light images are used in Figures S7 and S8, Supporting Information), since it is generated simultaneously with the retardance image, albeit small amount of error might occur due to small number of saturated pixels incorporated by the polarization. It is observed that the elliptical polarization provides a better contrast of the nanoplatelets and flakes by permitting depth‐resolved imaging^[^ ^26^ ^]^ in the turbid media (Figure S12, Supporting Information). This method is applicable to other anisotropic 2D materials to the same extent regardless of their various degree of birefringence (Δ*n*). Layered materials may possess a wide range of birefringence such as black phosphorus (BP) has a Δ*n* of ≈0.245, rhenium disulfide (ReS_2_) has a Δ*n* of ≈0.037, and rhenium diselenide (ReSe_2_) has a Δ*n* of ≈0.047 at 520 nm wavelength.^[^ ^27^ ^]^ Since, this method normalizes the principal components (brightness and retardance) between 0 and 1 by extracting them from the single‐channel 2D feature images and uses the normalized data as input to the machine learning process, a change in Δ*n* will not affect the quantification process. After the quantification process, the relevant birefringence value should be used for estimating the thickness.

## 4 Conclusion

There is a general lack of methods to characterize and quantify the liquid‐phase exfoliated sheets, nanoplatelets and flakes of graphitic materials. The importance and urgency of detecting and quantifying them have been highlighted in recent articles. We demonstrated that the LPE‐processed bulk liquid samples could readily be imaged, and their optical phase retardation can precisely be quantified with polarization of light. The quantified brightfield and cross‐polarized optical features are processed through unsupervised machine learning algorithms and high throughput statistics for measuring quantities of the highly absorbing flakes, the partially exfoliated nanoplatelets and the well‐exfoliated 2D sheets as three distinct clusters. We calculated the percentages of each species and estimated their lateral sizes, thicknesses, and concentrations through pixel‐by‐pixel analysis. This combination of qPOM and clustering method can be an efficient in situ classification and quantification tool during industrial‐scale production. The theme of this clustering process was to remain fully unbiased and unsupervised and learn about the intrinsic classes and quantities of the exfoliated graphene materials when dispersed in a liquid. As we developed this method and learned about the classes and quantities of these materials in this research, we are confident that large sets of data can be trained by taking into account additional features such as pixel coordinates paving the way for more automated methods—it will be the subject of our future research.

## 5 Experimental Section

##### Materials

Graphite oxide (GtO) was purchased from Sixth Element Inc. (China) which was used to produce samples (1–9) with an initial solid fraction of 0.2 wt%. The liquid samples were dried for a week in a food dehydrator via natural evaporation and then kept in a vacuum desiccator for a week before using for the exfoliation process. The dried GtO films were exfoliated in water by using a high shear laboratory mixer, Silverson‐L4RT, as described by Paton et al.^[^ ^3^ ^]^ Different speeds (1000, 2000, and 5000 rpm) were applied for different periods during the exfoliation (sample 1–5). It is estimated that 1000 rpm is equivalent to a magnitude of 5000 s^−1^ shear rate. Ultrasonication (E‐Chrom Tech, 20 kHz) was also employed as a second method for various periods (samples 6–9). Seven graphene samples and one graphene oxide sample were purchased from various commercial companies of Asia, Europe, and Australia.

##### Atomic Force Microscopy

The atomic force microscopy was conducted in a JPK Nanowizard 3 AFM. The LPE‐processed samples were spin‐coated (Laurell technologies, WS‐400BZ‐6NPP/LITE) onto microscopic glass slides for imaging using Bruker NCHV cantilevers in alternating contact mode in the air.

##### Imaging Principle

LC‐PolScope (described in Figure S9 in the Supporting Information) enabled fast and label‐free quantification of the optical retardance and slow‐axis orientation of birefringent materials at the highest resolution. In this method, neither the specimen nor any microscope parts were required to be rotated or moved mechanically during imaging. Here, a universal compensator comprising a linear polarizer and two‐variable retarder plates namely LC‐A and LC‐B replaced the traditional compensator. The retarders were uniformly birefringent liquid crystal plates and their optical properties were controlled via electrooptical devices. In a typical measurement, both circular and elliptical illuminations of the specimen were generated from the predetermined retarder settings of LC‐A and LC‐B. The CCD camera captured the raw images of the specimen which was processed by an algorithm to generate a retardance and a slow‐axis orientation map of the specimen. This system was first developed at the Marine Biological Laboratory, Woods Hole, USA by Dr. Rudolf Oldenbourg.

##### Image Acquisition

The liquid samples were imaged in both traditional microscopic slides and rectangular capillary cells (0.2 × 2 × 75 mm). The samples were mixed properly by gentle shaking before loading for imaging. In the slides, the samples were loaded in between two coverslips and then another slip was placed on the top of the sample. A calibration procedure was followed before imaging the samples, where the background retardances were removed in an automatic process by manually capturing a background image in the sample‐free area on the mounted specimen. For a particular area of interest in the specimen, the microscope generated one circularly polarized, and four elliptically polarized light images. An algorithm computed the retardance, and the slow‐axis orientation image from these raw images instantaneously. For brightfield measurement, both the polarizer and the analyzer were removed from the optical train by sliding the holders. After adjusting the intensity of light, a brightfield image was captured for the same area of interest. A monochromatic illumination (546 nm) was used and the exposure time was kept constant throughout all imaging. The measured retardances were in the range of 0.1–273 nm. At least five measurements were conducted for each sample. From each measurement, a set of three micrographs was used in the quantification process—1) a brightfield micrograph, which contains photon absorption information, 2) a cross‐polar‐processed retardance micrograph, which contains phase retardation information, and 3) a cross‐polar‐processed slow‐axis micrograph containing both the phase retardation and orientation information.

Brightfield images were taken on the sample with the brightfield set‐up of the microscope, where both the polarizer and analyzer were removed from the optical path. The information is encoded in an 8‐bit TIFF image file, in HSV encoding. The value (V) channel contains the photon absorption information which is one of the features used for the quantification process.

The retardance image was taken with an LC‐PolScope attachment. The information was encoded in an 8‐bit TIFF image file, in HSV encoding. The V channel contains the retardance information which is the measure of the phase shift of two circularly polarized orthogonal lights passing through the sample. This is another feature used for the quantification process.

Slow‐axis information is complementary to the retardance information. The image was encoded in a 24‐bit TIFF image file, in HSV encoding. The Hue (H) channel represents the relative angle of orientation of the slow‐axis throughout the sample. The V channel contains the retardance value similar to the retardance image. The Saturation (S) channel does not contain any meaningful information. The clustered areas were specified on this image and used as a validation tool of the clustering process. For example, the slow‐axis orientation of a single particle or flake is constant and defines its size, whereas for the self‐aligned sheets it defines the liquid crystal domain.

##### The Unsupervised Machine Learning Method

Clustering the LPE‐processed materials based on their optical features into the similar category without taking prior knowledge about these materials into consideration is the goal of the clustering process. This fundamental approach will enable the pixel‐by‐pixel understanding and quantification of the whole LPE process. Firstly, an unsupervised *K*‐means algorithm was applied on the optical features acquired via the LC‐PolScope system. The LPE‐processed materials were treated as *n* number of observations or pixels distributed in a 2D feature matrix (*n* × *2*). The two feature dimensions are brightness values and retardance values.

Assuming the data {*x_i_*}, *i* = 1, 2, ..., *n* to be a set of *n* 2D pixels grouped into a set of *k* clusters, *C* = {*c_k_*, *k* = 1, 2, …, *K*}. If *μ*_*k*_ is the mean of cluster *c_k_*, *K*‐means algorithm partition the data in a way that the squared Euclidean distance or error measure between the *μ*_*k*_ and the pixels within the cluster, *c_k_* defined as $\sum_{x_{1}\in c_{k}}{\left(x_{i}-\mu_{k}\right)}^{2}$ is minimized. The goal of *K*‐means is to minimize this error measure $W_{k}=\sum_{k=1}^{K}\sum_{x_{1}\in c_{k}}{\left(x_{i}-\mu_{k}\right)}^{2}$ over all clusters. Since the error measure decreases monotonically with the increase of *K*, finding an optimal *k* is a notoriously challenging part of the clustering process.

##### The Optimal Number of Clusters

Several heuristics approaches were proposed for determining the optimal number of clusters—here Gap‐statistics method was applied due to its versatility and effectiveness. The Gap is defined as the numerical difference between the experimental or observed error function and the expected error function of uniformly distributed null reference data. In this method, both the error measures were recorded to observe the elbow where the Gap is maximum indicating the optimal number of clusters in the data set.

##### The Gap‐Statistics Process

Initially ten random value feature matrices were generated for measuring the Gap. The values were randomized between the minimum and maximum values of observation in each dimension. The random feature matrices were fitted in *K* initial number of clusters and the mean of all the calculated inertia (*W_k_*) was calculated. This is the expected inertia. The Gap‐statistics is generated for a random number of initial clusters ranging from 1 to 100 (Figure S3, Supporting Information). Similarly, the mean inertia (*W_k_*) of the input feature matrix was calculated. As mentioned earlier, the error measure (*W_k_*) decreased monotonically with the increase of the initial cluster number. The optimal number of clusters is corresponding to that *k* where the observed inertia, log (obs − *W_k_*) has fallen the farthest below the expected inertia, log (exp − *W_k_*). Therefore, the optimal *k* is corresponding to the highest point in the inertia Gap, (log (exp − *W_k_*) − log (obs − *W_k_*)) versus *K* curve. Different statistical criterions such as the first local maxima, and global maxima in the Gap method were considered for determining the optimal number of clusters in the data set.

##### Statistical Analysis

Single‐channel 2D feature images were used and the principal components (brightness and retardance) were extracted as input data. The data were then normalized such that all values lie between 0 and 1 using “img_as_float” function of scikit‐image library. Statistical analysis was conducted to find the presence of clusters in the input data, optimum number of clusters, percentage of materials in each cluster, minimum, maximum, mean, median, and standard deviation of principal components in each cluster. To do these statistical analyses, an unsupervised statistical learning method widely known as K‐means^[^ ^20^ ^]^ from scikit learn library was employed to find Euclidean distance based similar features among the data sets in an iterative method. An error function based on Gap‐statistics was generated^[^ ^22^ ^]^ and concomitantly compared with an appropriate null reference distribution of the original pixel data to determine the optimum cluster. The error function is the sum of squared Euclidean distances around the random cluster‐means. The null reference distribution is statistically designed based on the hypothesis that if there are no subgroups present in the pixel data set, the observed or experimental error function will follow the null or expected function.^[^ ^22^ ^]^ The distribution was generated so that the minimum and maximum values of distribution correspond to the minimum and maximum values of feature matrices that are tested. The distribution is uniform within this range, zero elsewhere. The maximum Gap between the two functions indicates the optimal number of clusters in a data set.^[^ ^23^ ^]^ 1‐standard‐error, local and global error measures were used to determine whether the clusters are well‐separated from each other or not.^[^ ^21^ ^]^ As relative clustering validation, the initial number of assumed cluster‐means was changed to test its influence on the optimal number of clusters. For additional clustering validation, the clustering results were manually compared with the input images as well as by conducting AFM and elliptical polarization experiments. The possibility of subgroups was tested within the clusters by the Gap‐statistics method. The algorithm generated the mean, median and standard deviation (SD) of the principal components of each cluster, which were analyzed in Excel to estimate the relative proportions of different species. The distribution of lateral size and thickness of the species was plotted using Origin's distribution function.

##### Code Availability

The algorithm utilized in this research article is available on request from TB (titon@vimmaniac.com) or MM.

##### Data Availability

All relevant data generated in this study are included in the article and/or its supplementary information files. Additional raw data can be requested from M.M.

## Conflict of Interest

The authors declare no conflict of interest.

## Author Contributions

M.J.A. and M.M. conceived the project. M.J.A. designed and conducted the experiments and analyzed the data. T.B. and M.J.A. developed the algorithm. M.J.A., T.B., M.S., and M.M. wrote the manuscript. All authors read and approved the final version of the manuscript.

## Supporting information

Supporting Information
