# Supplementary material for: A High Throughput and Unbiased Machine Learning Approach for Classification of Graphene Dispersions
Source: Adv Sci (Weinh). 2020 Aug 25;7(20):2001600. doi: 10.1002/advs.202001600 (PMC7578897; doi:10.1002/advs.202001600)
Supplement: Supplementary file 1 — Supporting Information [file ADVS-7-2001600-s001.pdf]

© 2020 Wiley-VCH GmbH

ADVANCED SCIENCE
Open Access

Supporting Information

for *Adv. Sci.*, DOI: 10.1002/advs.202001600

A High Throughput and Unbiased Machine Learning Approach for Classification of Graphene Dispersions

*Md. Joynul Abedin, Titon Barua, Mahdokht Shaibani, Mario Boley, and Mainak Majumder**

A High Throughput and Unbiased Machine Learning Approach for Classification of Graphene Dispersions

*Md. Joynul Abedin, Titon Barua, Mahdokht Shaibani, Mario Boley, and Mainak Majumder**

M.J. Abedin, Dr. M. Shaibani, Prof. M. Majumder
Nanoscale Science and Engineering Laboratory (NSEL)
Department of Mechanical and Aerospace Engineering
Monash University
Clayton, VIC 3800, Australia
E-mail: mainak.majumder@monash.edu

M.J. Abedin, Prof. M. Majumder
ARC Research Hub on Graphene Enabled Industry Transformation
Monash University
Clayton, VIC 3800, Australia

T. Barua
Vimmaniac Ltd.
83/A, Sugandha, Panchlaish, Chittagong 4203, Bangladesh

Dr. M. Boley
Department of Data Science & AI
Monash University
Clayton, VIC 3800, Australia

Copyright WILEY-VCH Verlag GmbH & Co. KGaA, 69469 Weinheim, Germany, 2018.

Supporting Information

A High Throughput and Unbiased Machine Learning Approach for Classification of Graphene Dispersions

*Md. Joynul Abedin, Titon Barua, Mahdokht Shaibani, Mario Boley, and Mainak Majumder**

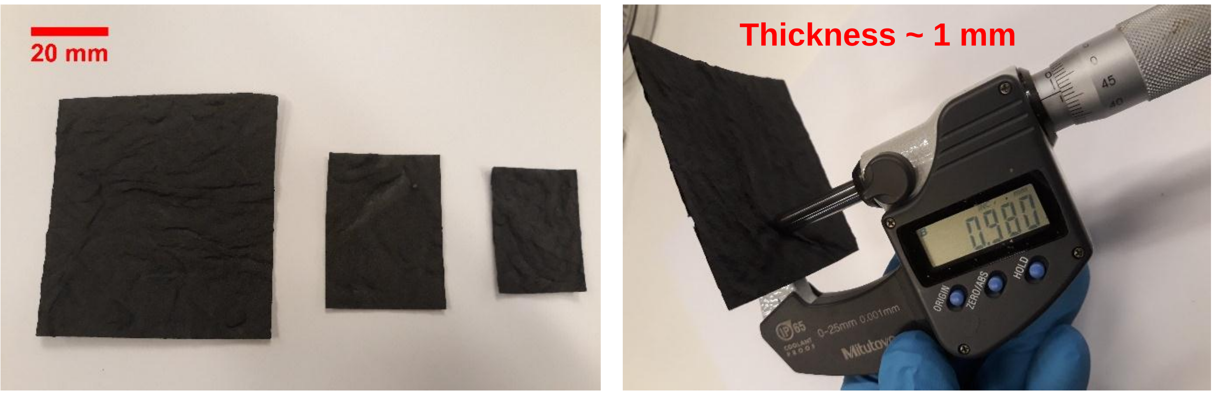

Figure S1. The thickness of the graphite oxide film that has been used in the LPE process.

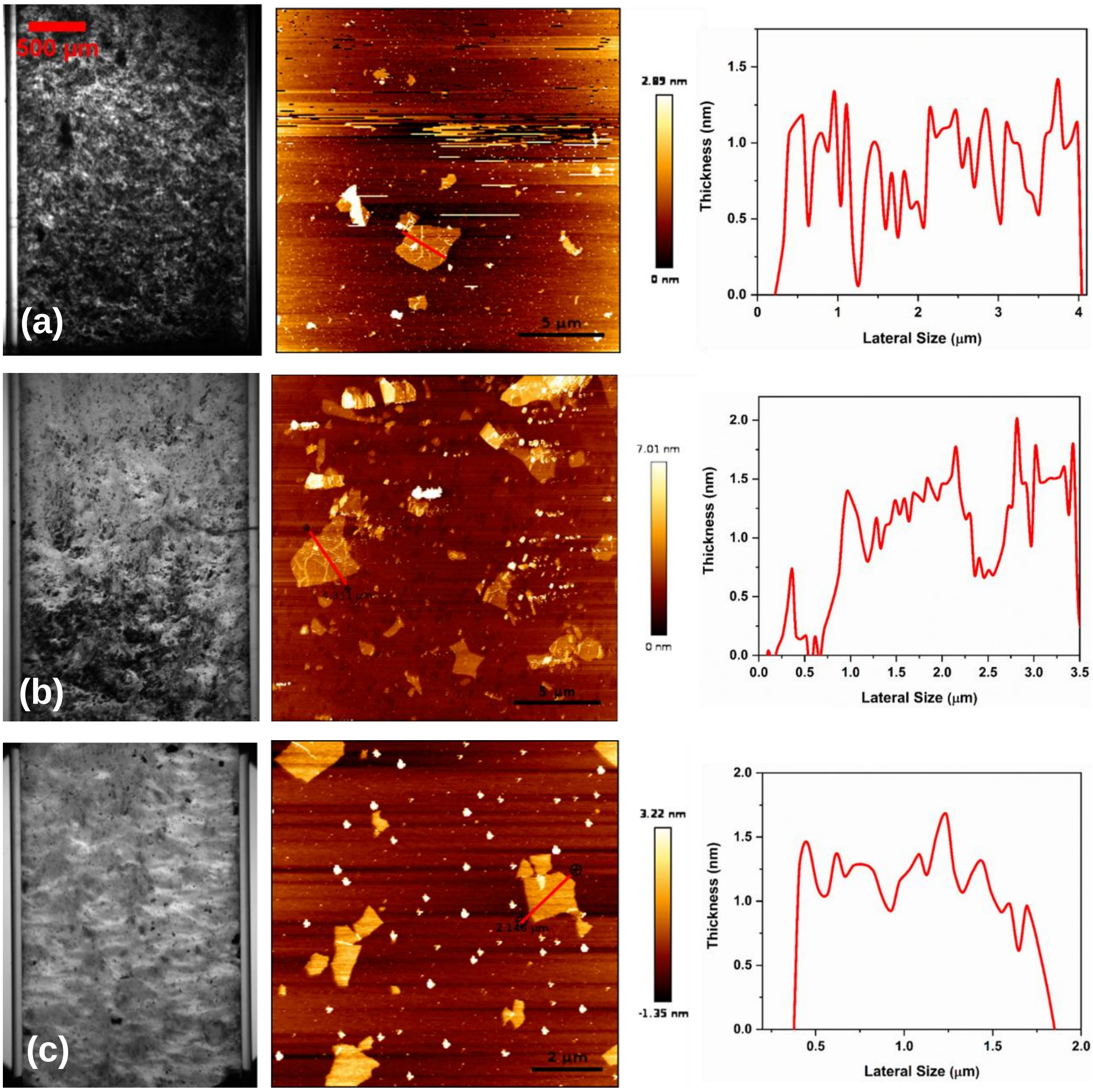

Figure S2: Demonstration of different degrees of exfoliations as a function of the time of shearing. (left panel) Elliptically polarized light images revealed that samples produced at low-speed, (a) 1000 rpm contain a large quantity of un-exfoliated materials, and that quantity decreased as shearing speed was increased to (b) 2000 rpm and further when increased to (c) 5000 rpm. AFM imaging after recommended and established cleaning processes for sample preparation such as centrifugation and spin-coating is showing that single layers are produced regardless (middle & right panel).

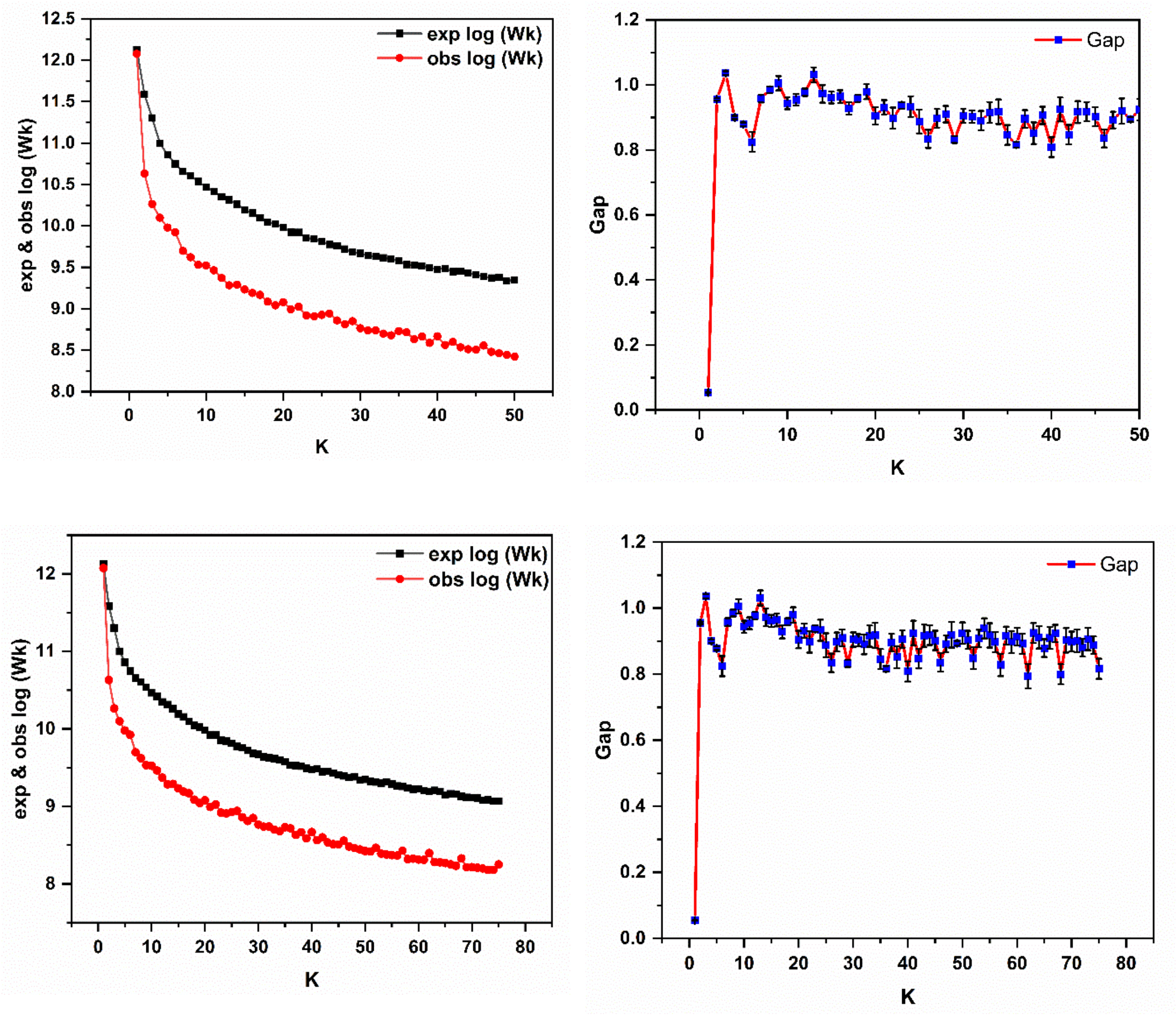

Figure S3. Optimal number of clusters for different initial assumptions of k. Both the local maximum and global maximum estimates similar optimal clusters in the data even though the number of assumed initial cluster is changed. This signifies that the clusters are well-separated. The error bar represents SD.

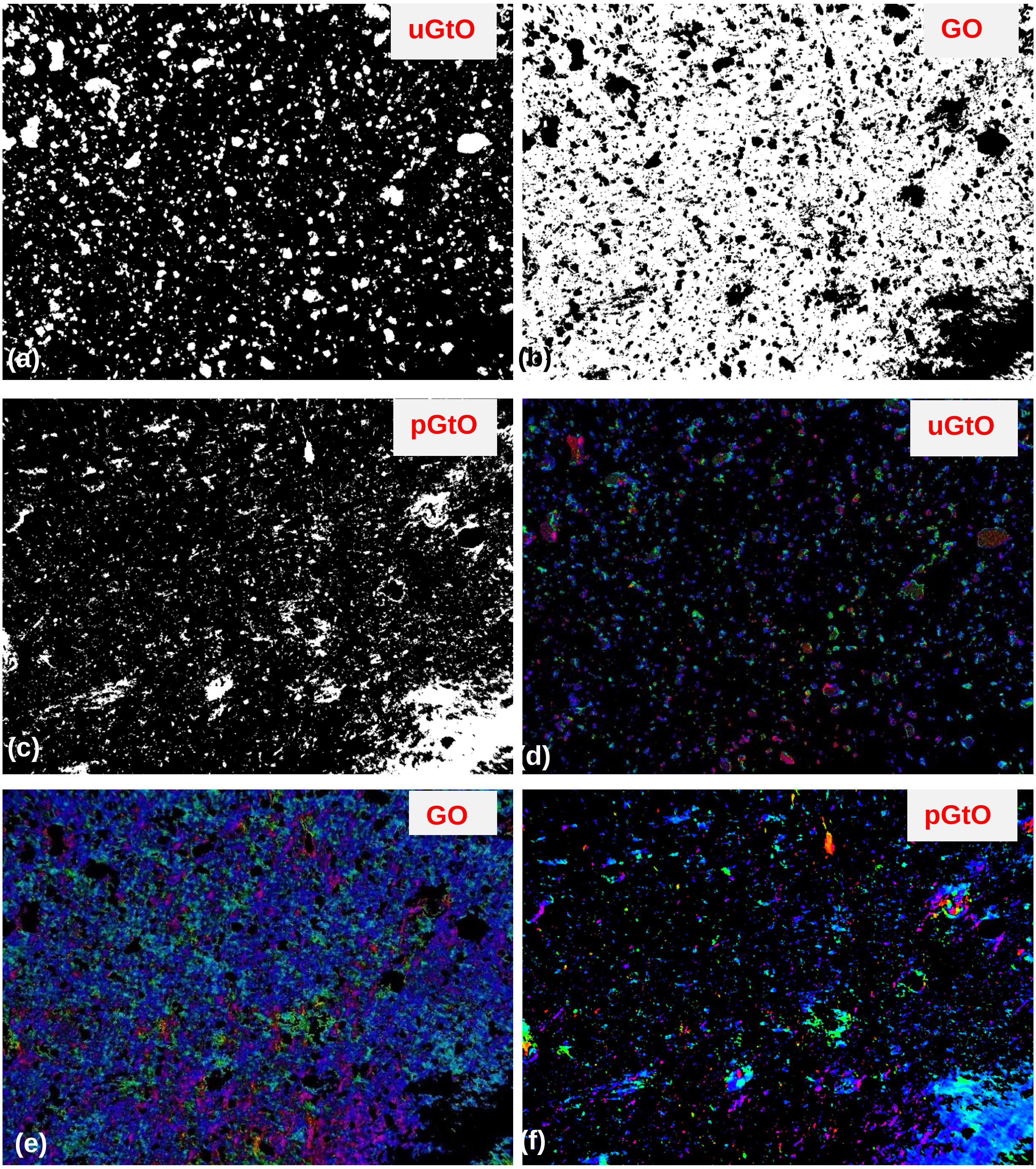

Figure S4. Binary (a-b-c) and slow-axis images (d-e-f) of clusters corresponding to Figure 2

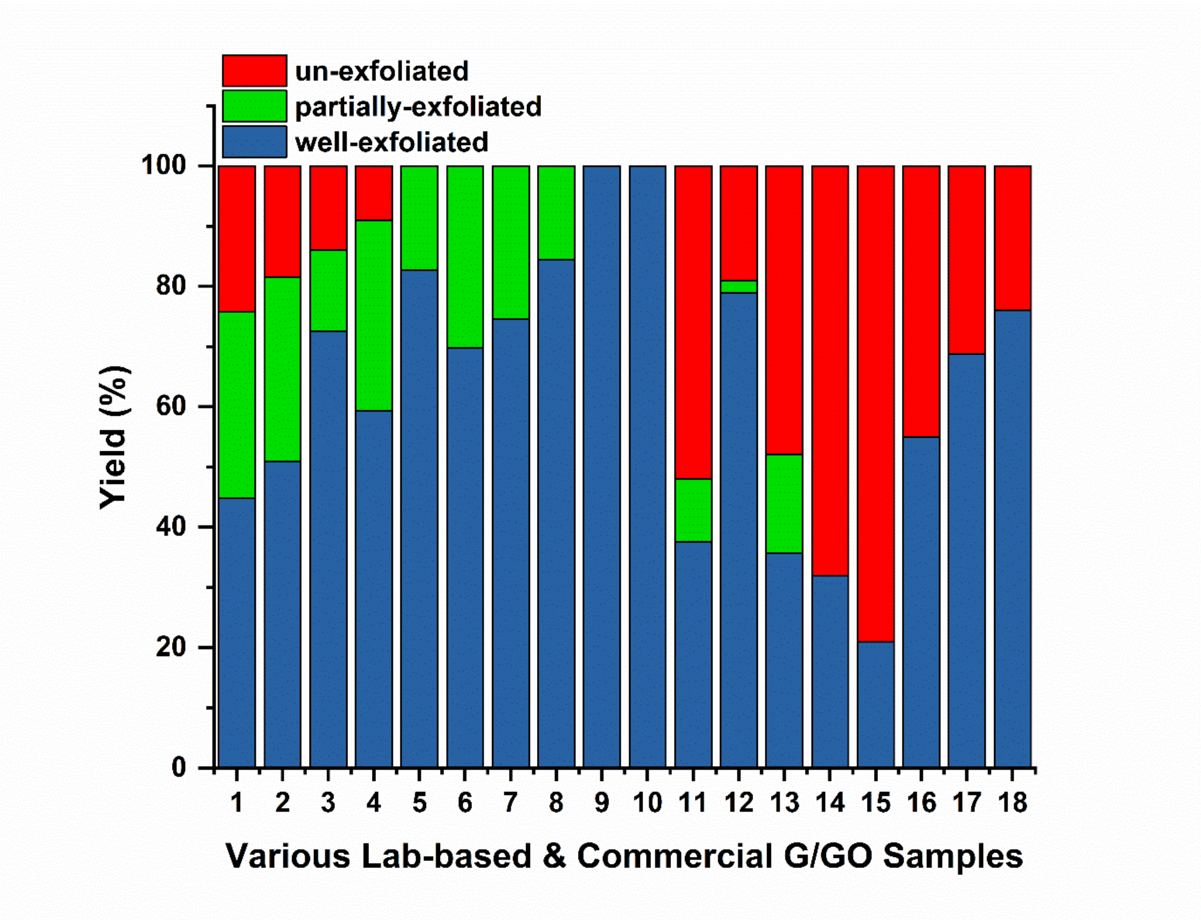

Figure S5. Estimated yield of G/GO samples produced *via* mechanical shear, ultrasonication and purchased from commercial companies.

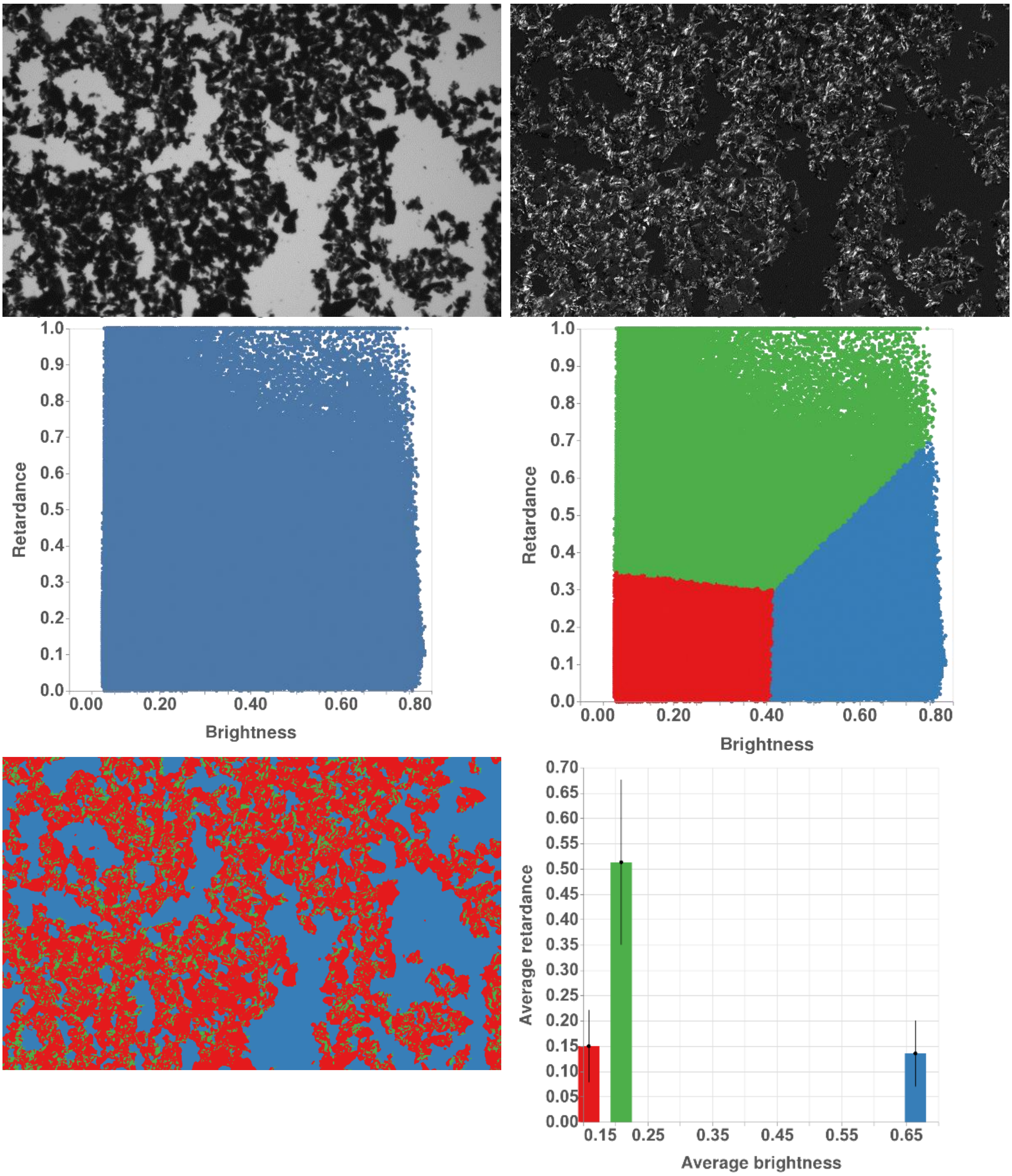

Figure S6. Results of commercial graphene sample 11. The error bar represents SD of retradance.

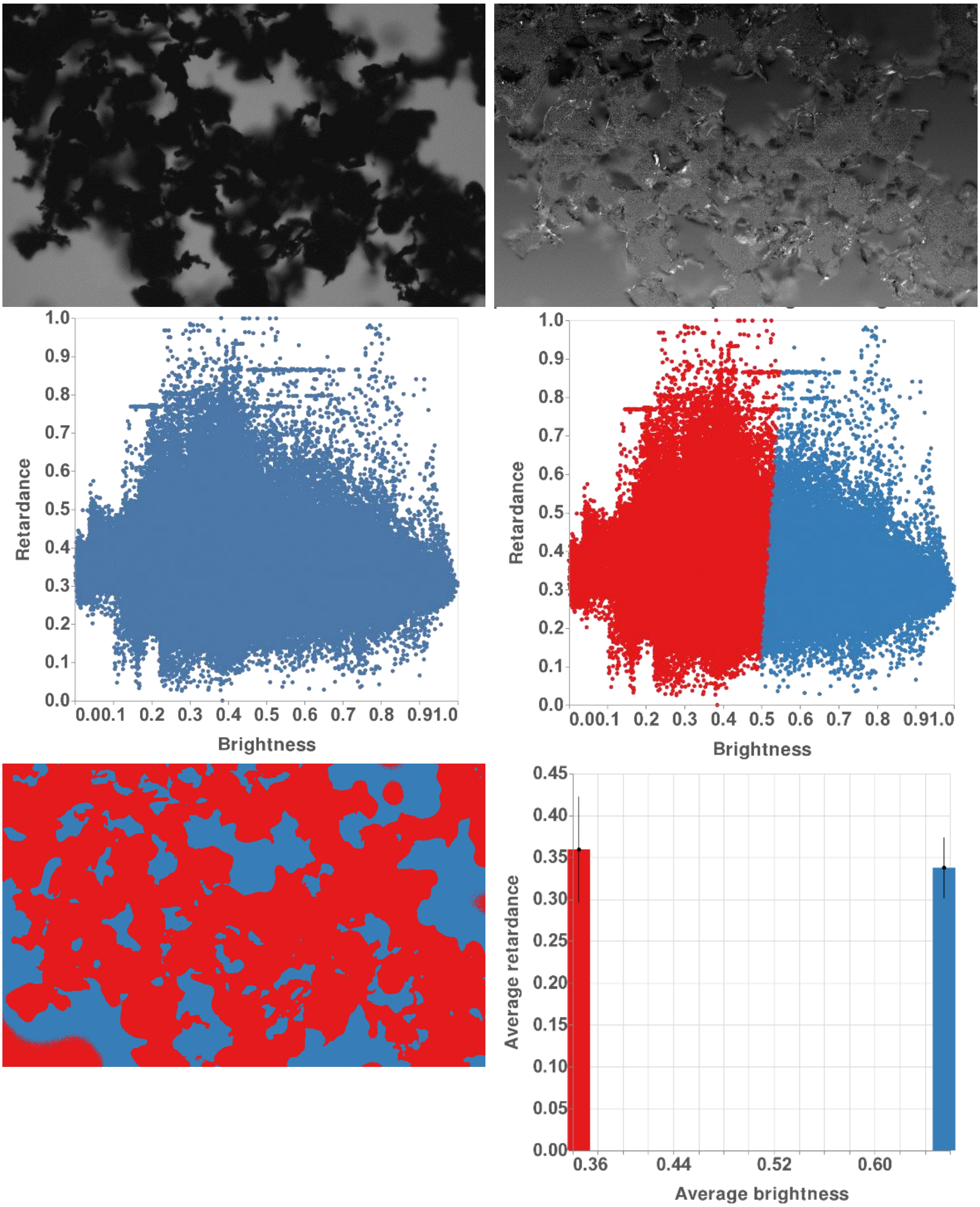

Figure S7. Results of commercial graphene sample 14. The error bar represents SD of retradance.

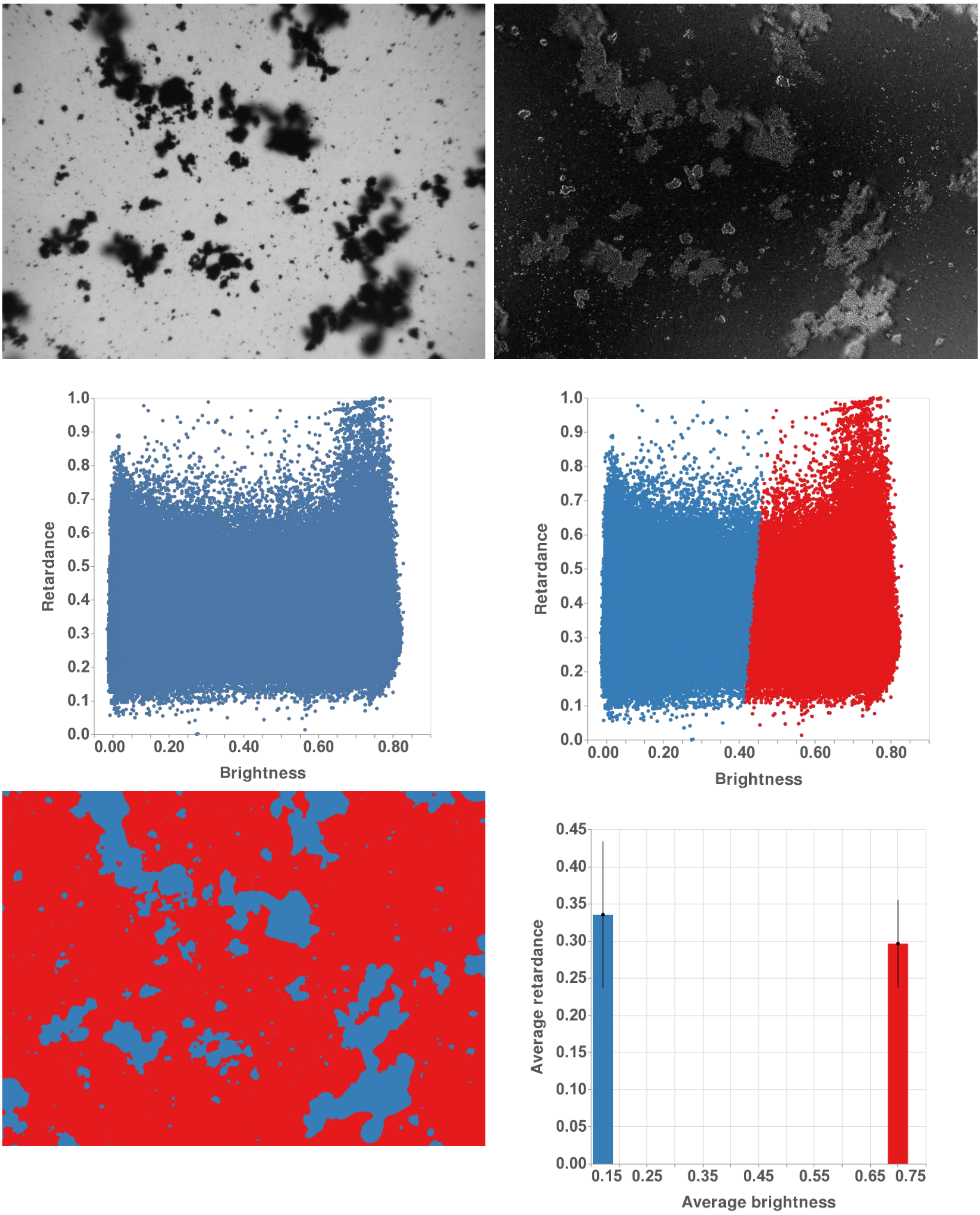

Figure S8. Results of commercial graphene sample 18. The error bar represents SD of retradance.

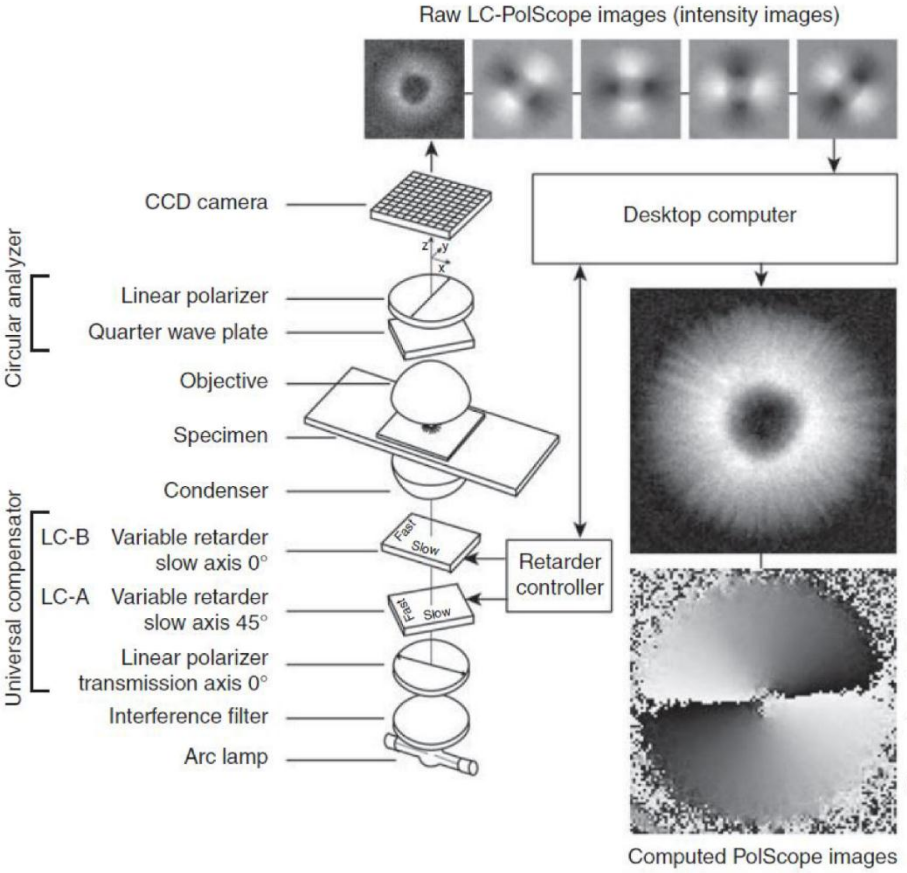

Figure S9. The principle of LC-PolScope system. One circularly polarized light image (first from left at the top row) and four elliptically polarized light images (second to fifth image) are generated for each specimen (an example of birefringent aster isolated from surf clam egg is given) by changing the retardance settings of LC-A and LC-B. An image processing algorithm synchronizes the whole process and computes the retardance image within a few seconds. Simultaneously, it generates a slow-axis orientation map of birefringent material from the raw images as shown in the right panel. (reprinted with permission from Dr. Rudolf Oldenbourg by co from the OpenPolScope website[1])

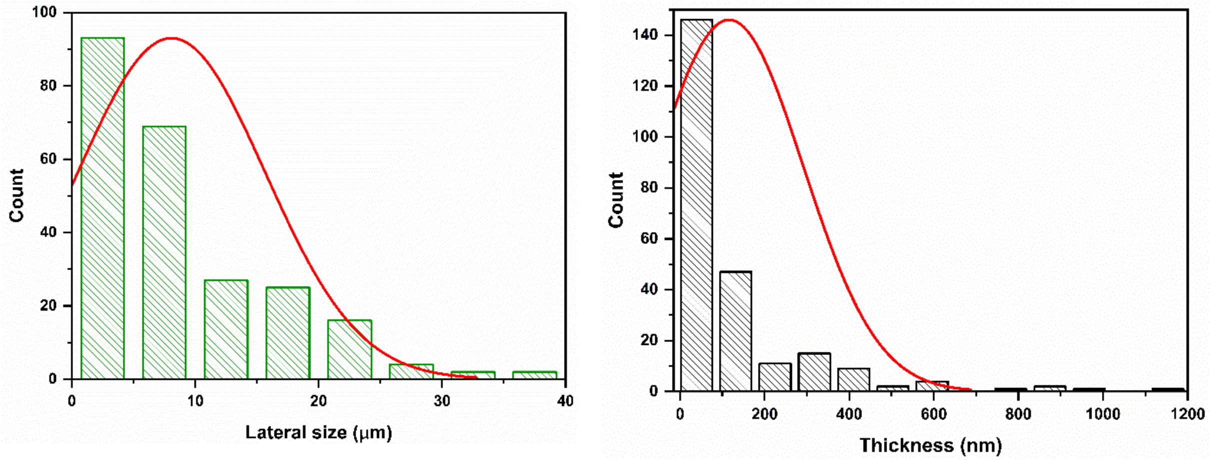

Figure S10. Lateral size and thickness distribution of nanoplatelet and 2D sheet, calculated by analysing the intra-cluster data of sample 1. The solid lines are the normal fits of the data. Skewness in the distribution indicates low-speed processed sample contains 2D sheets as well as large flakes in the dispersion.

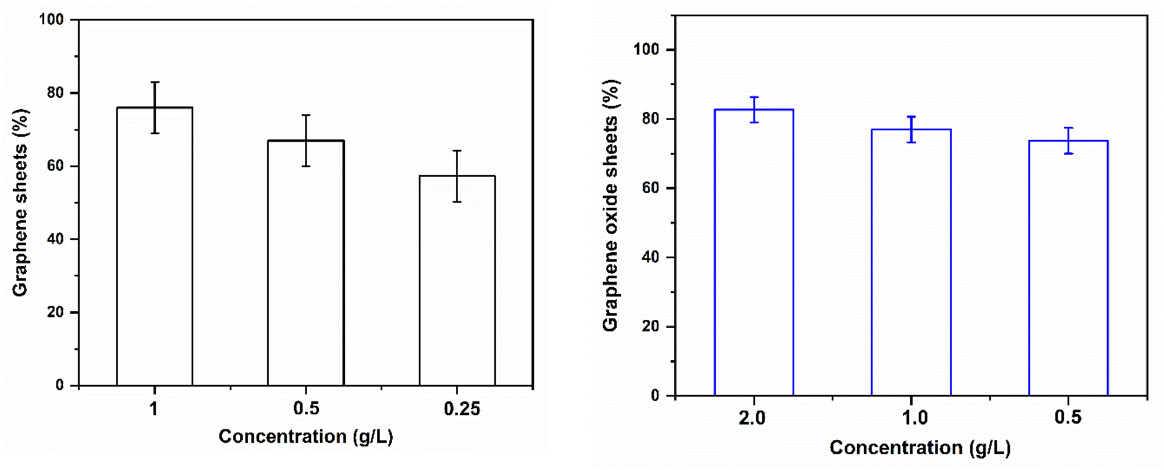

Figure S11. The samples [GO (sample 5) and G (sample 18)] can be processed at different concentrations. The error bar represents SD which is calculated from three sets of clustering data for each concentration.

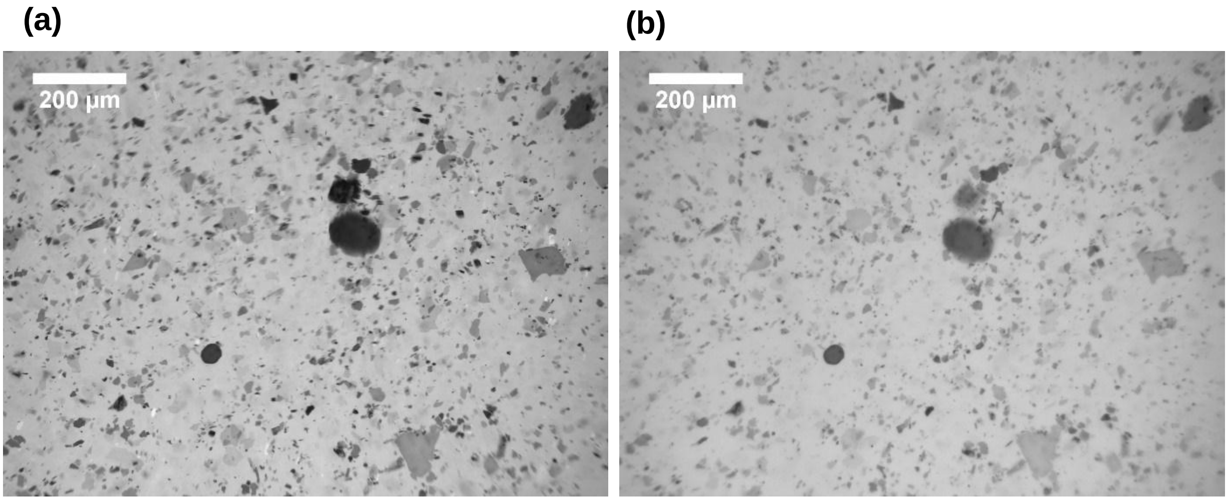

Figure S12. Elliptically polarized light image (a) provides better contrast of the nanoplatelets and flakes compared to brightfield image (b), and can alternatively be used in the quantification process.

Reference

[1] R. Oldenbourg, S. Mehta, A. Verma, G. Harris, Vol. 2019, 2019.
